# Digesting dietary miRNA therapeutics

**DOI:** 10.18632/oncotarget.4357

**Published:** 2015-06-08

**Authors:** Mark Yarmarkovich, Kendal D. Hirschi

**Affiliations:** Children's Nutrition Research Center, Baylor College of Medicine, Houston, TX, USA

Hippocrates famously advised, “Let food be thy medicine and thy medicine be thy food.” Numerous plant-derived compounds are used as cancer therapeutics including antimitotics, topoisomerase inhibitors, and kinase inhibitors. Here we will review emerging evidence suggesting that diet derived small RNAs may be an inexpenisive, non-invasive and affordable way to deliver cancer treatments.

RNA interference (RNAi), the use of short double-stranded RNA sequences to silence endogenous genes is emerging as the next generation of therapeutics. A well-designed RNAi molecule is able to differentially silence a mutated oncogene with just a single mutated base, leaving the wild-type unaffected. Further, due to the use of intrinsic cellular mechanisms for gene silencing, RNAi exerts an amplified stoichiometric effect, suppressing expression of thousands of mRNA transcripts per RNAi molecule. The challenge with the use of RNAi therapeutics remains their efficient uptake and transportation to target cells. Various delivery techniques are being investigated including viral delivery, engineered nanoparticles and modified nucleic acid chemistry. Each method faces a unique set of challenges including efficiency, safety, and immunogenicity. Utilization of therapeutics by the oral route promises low costs, minimal invasiveness, and high compliance, making this the holy grail of delivery methods.

In 2012, a study demonstrated a dietary microRNA (miRNA) from rice was packaged and systemically circulated in consuming mice to silence a liver gene [1]. Plant miRNAs contain a unique 2′-O-methylation on the ribose of the 3′ nucleotide, which was used to distinguish the plant-derived miRNAs from endogenous miRNAs. The authors postulated that this modification promotes stability of dietary small RNAs. In the last several years, numerous reports have failed to establish dietary delivery of miRNAs as a general means of gene regulation in healthy consumers [2]. However, our group has reported dietary regimes and pharmacological methods to enhance detection of dietary miRNA in mice serum [3]. Our work implies that high doses and certain gut pathologies enhance uptake of dietary small RNAs. In other studies, mice fed large doses of synthetic plant-modified miRNAs appear to have therapeutic potential [4]. Using a mouse model of colon cancer, a cocktail of tumor-suppressing miRNAs with plant-based chemistries were fed to mice and significantly decreased tumor burden. We posit this therapy was successful due to the enhanced uptake capabilities in the impaired gut. Additional studies have shown that oral administration of high dosages of small RNAs halt the replication of the influenza virus in mice [5]. Taken together, these studies demonstrate that particular diets and consumers are amenable to dietary miRNA cancer therapeutics.

Cells are known to release small vesicles (exosomes and microvesicles) from their membranes, packaging and protecting their contents (mRNA, protein, membrane contents, and miRNAs). Though the biological role of these vesicles is still being explored, the packaging of specific subsets of cellular molecules suggests that exosomes function as mediators of systemic intercellular communication. Recent examples have also suggested that exosomes are able to cross barriers between species. Reports have described the detection of cow's milk-derived miRNAs in serum of the consumers at physiologically relevant concentrations [6]. In mouse feeding experiments, these milk-based exosomes protect dietary miRNAs and facilitates their regulatory capabilities in the consumer. Studies from other groups have shown ‘plant exosomes' isolated from fruits and vegetables can be taken up by macrophages and monocytes and act on inflammatory pathways. In addition to naturally occurring mechanisms of cross-talk, orally delivered bacterial cells expressing short hairpin RNA (shRNA) have entered clinical trials and orally administered nanoparticles loaded with small interfering RNA (siRNA) have shown systemic gene silencing in macrophages [7]. These findings suggest that carriers from particular foods, especially milk, may serve as efficient vehicles for delivery of therapeutic molecules.

The future of dietary miRNAs as therapeutics requires new tool development and mechanistic insights regarding uptake and delivery. Dietary miRNA studies are confounded by high variation and low copy number detection. These issues need to be addressed with judicious and standardized detection methods. Use of sensitive tissue sensors will help identify both the presence and functionality of low-level absorbed miRNAs. Dietary RNAi function in *C. elegans* has been well described and transporters for these small RNAs characterized. Animal genomes contain sequences that are homologous to those responsible for the systemic RNAi effect seen in *C. elegans*. Could we impact dietary miRNA uptake by altering the localization and kinetics of these putative endogenous transporters? We favor a model where therapeutic plant-based miRNAs within the plant matrix display enhanced bioavailability compared to synthetic plant-based miRNAs. Scientists are in the process of expressing therapeutic miRNAs in crops plants to assess efficacy of these engineered foods fed to patients.

In addition, the milk-based approach to therapeutic miRNA delivery may prove especially useful in infants. The developing fetus and newborns may lack acquired ‘protective' mechanisms in the gut that deter small RNA uptake in adults. Perhaps nature has already provided an ideal mechanism for the safe and efficient delivery of next generation therapeutics, allowing us to rekindle the notion of using foods as medicine.
